# P-1415. Social Determinants of Health and Side Effects Significantly Predict Pre-Exposure Prophylaxis (PrEP) Preferences for Every 2 Month Long-Acting Injectable vs. Daily Oral PrEP among Cisgender Women in the United States and the Dominican Republic

**DOI:** 10.1093/ofid/ofae631.1590

**Published:** 2025-01-29

**Authors:** Deanna Kerrigan, Rachel Scott, Allison O’Rourke, Noya Galai, Tahilin Sanchez Karver, Wendy W Davis, Aimee A Metzner, Alan Oglesby, Patricia Moriarty, Tamara Taggart, Clare Barrington, Hoisex Gomez, Martha Perez, Yeycy Donastorg

**Affiliations:** George Washington University, Washington, District of Columbia; MedStar Health Research Institute, Washington, DC; George Washington University, Washington, District of Columbia; John Hopkins Bloomberg School of Public Health, Baltimore, Maryland; Johns Hopkins University, Baltimore, MD; George Washington University Milken Institute School of Public Health, Washington, District of Columbia; ViiV Healthcare, Durham, North Carolina; ViiV Healthcare, Durham, North Carolina; MedStar Washington Hospital Center, Washington DC, District of Columbia; George Washington University, Washington, District of Columbia; UNC Gillings School of Global Public Health, Chapel Hill, North Carolina; Unidad de Vacunas e Investigación IDCP, Santo Domingo, Distrito Nacional, Dominican Republic; Instituto Dermatologico y Cirugia de la Piel, Santo Domingo, Distrito Nacional, Dominican Republic; INSTITUTO DERMATOLOGICO Y CIRUGIA DE PIEL "DR. HUBERTO BOGAERT DIAZ", SANTO DOMINGO, Distrito Nacional, Dominican Republic

## Abstract

**Background:**

Significant HIV inequities exist among cisgender women (CGW) across settings. Black CGW in the United States (US) have an HIV diagnosis rate 10.8 times greater than White CGW. Female sex workers (FSW) have 30-times increased odds of acquiring HIV vs. CGW globally. HIV disparities and low pre-exposure prophylaxis (PrEP) uptake in CGW are driven by social determinants such as stigma. As long-acting injectable (LAI) PrEP rolls out, it is critical to understand CGW’s PrEP preferences.

**Figure:**
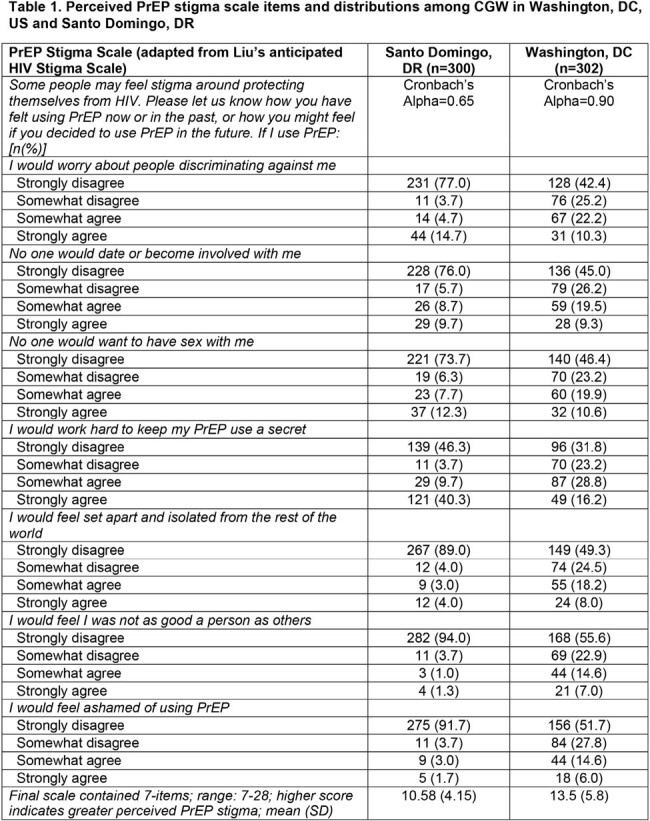

**Methods:**

Cross-sectional surveys were conducted among CGW who were: ≥18 years and HIV- in Washington, DC, US (n=302) and Santo Domingo, Dominican Republic (DR) (n=300). US CGW [86.4% Black; 30 yrs (mean) were recruited in reproductive health clinics. DR CGW [100% mixed race; 32 yrs (mean)] were FSW recruited by peer navigators. Stepwise multivariate logistic regression assessed demographic, behavioral and social determinants of PrEP preference (every 2-month LAI vs. daily oral) including aggregate measures of perceived PrEP stigma (**Table 1**) and PrEP medication optimization (**Table 2**) based on existing HIV scales.

**Figure:**
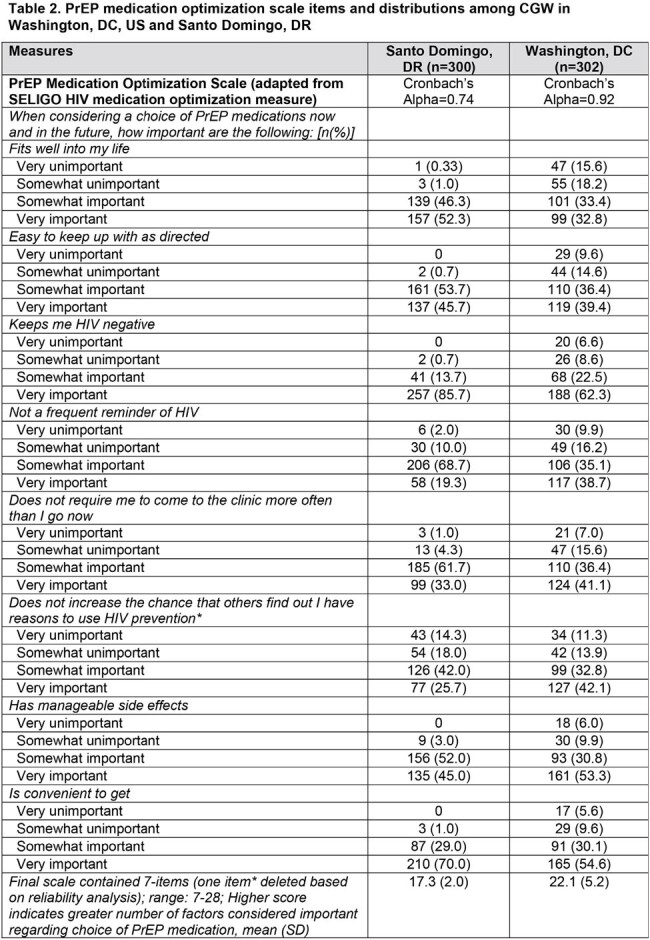

**Results:**

The majority of CGW preferred LAI vs. daily oral PrEP: 71.0% (DR); 73.2% (DC). In both settings (**Table 3**), LAI preference was inversely associated with whether they would be bothered by injection site pain or tenderness for a few days to a week (DC AOR 0.54; 95% CI 0.31-0.96; DR AOR 0.46; 95% CI 0.23-0.93). In DC, each additional item considered important in the PrEP optimization scale was associated with a greater odds of LAI PrEP preference (AOR 1.06; 95% CI 1.01-1.10). Having had an HIV test and PrEP stigma were marginally significant (p< 0.10) in DC. In DR, greater odds of LAI preference was associated with prior injectable medication or contraception experience (AOR 2.39; 95% CI 1.37-4.18) and greater alcohol use (AOR 1.90; 95% CI 1.11-3.25); lower odds of LAI preference was associated with PrEP stigma (AOR 0.93; 95% CI 0.88-0.99) and sexual partner violence (AOR 0.39; 95% CI 0.18-0.82).

**Figure:**
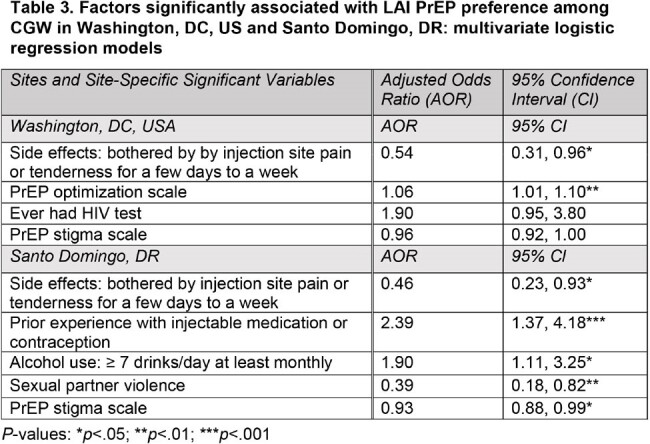

**Conclusion:**

Shared and context specific factors drive PrEP preferences among diverse groups of CGW. Social determinants and side effects management should be addressed in tailored interventions to ensure equitable PrEP uptake including LAI PrEP in CGW.

**Disclosures:**

**Deanna Kerrigan, PhD, MPH**, ViiV Healthcare: Grant/Research Support **Rachel Scott, MD,MPH,FACOG**, DHHS Perinatal Guidelines: Board Member|UW STD Prevention Training Center (UW STD PTC): Honoraria|ViiV Healthcare: Advisor/Consultant|ViiV Healthcare: Grant/Research Support|Vindico CME: Honoraria **Allison O’Rourke, MPH**, ViiV Healthcare: Grant/Research Support **Wendy W. Davis, EdM**, ViiV Healthcare: Grant/Research Support **Aimee A. Metzner, PharmD, AAHIVP**, GSK: Stocks/Bonds (Public Company)|ViiV Healthcare: Employee **Alan Oglesby, MPH**, GSK: Stocks/Bonds (Public Company)|ViiV Healthcare: Employee **Tamara Taggart, PhD, MPH**, HealthHIV: Honoraria

